# Misbehaviour of *XIST* RNA in Breast Cancer Cells

**DOI:** 10.1371/journal.pone.0005559

**Published:** 2009-05-15

**Authors:** Silvia M. Sirchia, Silvia Tabano, Laura Monti, Maria P. Recalcati, Manuela Gariboldi, Francesca R. Grati, Giovanni Porta, Palma Finelli, Paolo Radice, Monica Miozzo

**Affiliations:** 1 Department of Medicine, Surgery and Dentistry, Medical Genetics Unit, Università degli Studi di Milano, Milano, Italy; 2 Laboratory of Medical Cytogenetics and Molecular Genetics, Istituto Auxologico Italiano, Milano, Italy; 3 IFOM, Fondazione Istituto FIRC di Oncologia Molecolare, Milano, Italy; 4 Department of Experimental Oncology , Fondazione IRCCS Istituto Nazionale Tumori, Milano, Italy; 5 Cytogenetics and Molecular Biology Unit, Laboratorio TOMA, Varese, Italy; 6 Department of Experimental and Clinical Biomedical Sciences, Università dell'Insubria, Varese, Italy; 7 Department of Biology and Genetics for Medical Sciences, Università degli Studi di Milano, Milano, Italy; Health Canada, Canada

## Abstract

A role of X chromosome inactivation process in the development of breast cancer have been suggested. In particular, the relationship between the breast cancer predisposing gene *BRCA1* and XIST, the main mediator of X chromosome inactivation, has been intensely investigated, but still remains controversial. We investigated this topic by assessing XIST behaviour in different groups of breast carcinomas and in a panel of breast cancer cell lines both *BRCA1* mutant and wild type. In addition, we evaluated the occurrence of broader defects of heterochromatin in relation to *BRCA1* status in breast cancer cells. We provide evidence that in breast cancer cells BRCA1 is involved in *XIST* regulation on the active X chromosome, but not in its localization as previously suggested, and that XIST can be unusually expressed by an active X and can decorate it. This indicates that the detection of XIST cloud in cancer cell is not synonymous of the presence of an inactive X chromosome. Moreover, we show that global heterochromatin defects observed in breast tumor cells are independent of *BRCA1* status. Our observations sheds light on a possible previously uncharacterized mechanism of breast carcinogenesis mediated by XIST misbehaviour, particularly in *BRCA1*-related cancers. Moreover, the significant higher levels of *XIST*-RNA detected in *BRCA1*-associated respect to sporadic basal-like cancers, opens the possibility to use *XIST* expression as a marker to discriminate between the two groups of tumors.

## Introduction

X chromosome inactivation (XCI) occurs early during embryogenesis and *XIST* (X inactive specific transcript) is the key player of the X chromosome silencing [1], [2]. XCI begins with the expression of *XIST* and stabilization of its noncoding RNA transcript *in cis*, along the X chromosome that is destined for inactivation [3], [4]. Subsequently, the inactive X (Xi) acquires the typical features of heterochromatin: late replication, hypoacetylation of histones H3 and H4, methylation of histone H3 lysines 9 and 27, lack of methylation of H3 lysine 4, methylation of DNA CpG islands and concentration of the variant histone macroH2A1 [5]–[7]. These epigenetic modifications appear to act synergistically and, once established in the soma, the inactive state is clonally and stably maintained through the subsequent cell divisions [3]–[6].

There is a substantial body of evidences to reveal the occurrence of XCI alterations in breast cancer cells. Several authors noted that some aggressive breast tumors do not show a detectable Barr body, the cytological evidence of the Xi [8]–[12]. Ganesan and colleagues [13] reported the first evidence of a communication of the inactive X chromosome with the protein codified by *BRCA1*, a main highly penetrance gene predisposing to breast and ovarian cancer development [14]. These authors showed that *XIST* RNA concentration on the Xi is supported by the BRCA1 protein and suggested that in *BRCA1*-associated carcinomas the lack of X inactivation is a consequence of BRCA1 deficiency, an assertion reiterated by the same group in a more recent study [15]. Otherwise, we reported [16] that in breast cancer cells, in spite of the presence of two or more X chromosomes, none of them is functionally inactivated, irrespective of *BRCA1* status, and all the X chromosomes are copies of native active X (Xa).

Our findings were subsequently confirmed by Richardson and collaborators [17] in basal-like breast cancers (BLC). BLC are a distinct breast carcinomas subtype, accounting for the majority (∼70%) of BRCA1-associated cancers and ∼15% of sporadic ones [18]–[22]. They are high-grade, aneuploid, invasive ductal carcinomas that show expression of cytokeratins of the basal layer of breast epithelium and do not express estrogen and progesterone receptors and HER2 [17], [23], [24]. Richardson and colleagues [17] described Xi loss and Xa replication as a frequent and distinctive feature in both sporadic and *BRCA1*-associated BLC, whereas it was rarely detected in non-BLC.

Eventually, the role of BRCA1 in XIST localization on Xi was questioned by studies [25], [26] that reported the absence of a cytological overlapping between BRCA1 and Xi or XIST territory, despite a limited, but not exclusive, BRCA1 accumulation abutting Xi. In addition, in BRCA1 depleted normal and tumor cells and in BRCA1 reconstituted cells, the BRCA1 status did not closely correlate with XIST localization. Finally, Vincent-Salomon et al. [27] pointed out that *BRCA1*-related tumors show heterogeneity of the XCI status, likely due to a high degree of X chromosome instability. The Authors observed that this instability led not only to the loss but also to an increase in the number of Xi.

We investigated some aspects of this intricate matter by sounding out the supposed relationship between BRCA1 and XIST expression/localisation and by assessing the nuclear XIST behaviour in breast cancer cells. Finally, we evaluated the occurrence of broader defects of heterochromatin in breast cancer cells in relation to *BRCA1* status.

## Results

### High levels of *XIST* RNA in *BRCA1*-associated breast carcinomas

**Figure 1:**
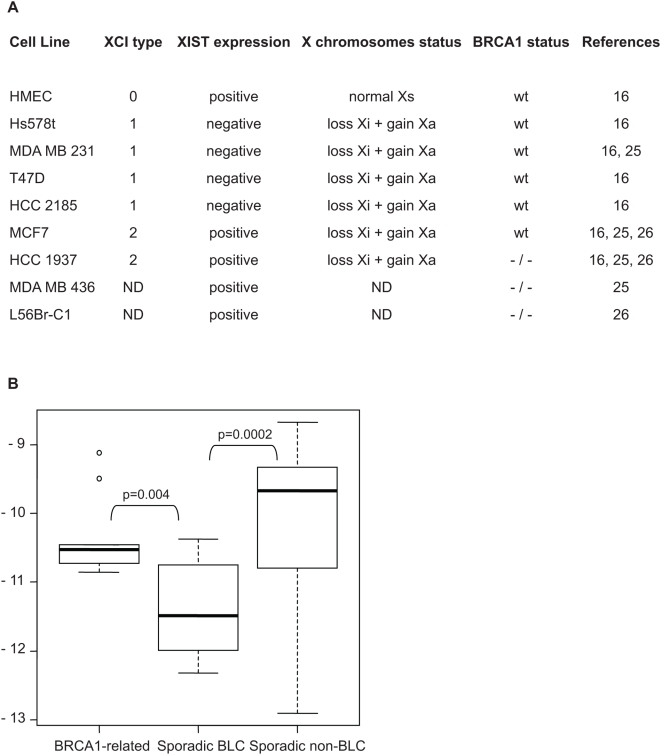
*XIST* expression and status of X chromosomes and *BRCA1* in HMEC and breast cancer cell lines, and evaluation of XIST levels in different groups of breast carcinomas. A) Classification of HMEC and breast cancer cell lines according to XCI type, based on the indicated X chromosome related features. *BRCA1* status is also reported. B) Box-plots of the log2-transformed amounts of *XIST* RNA measured by quantitative real-time RT-PCR in the indicated groups of primary human breast cancers. Each box-plot represents the first quartile (lower edge of the box), median value (bar inside the box), third quartile (upper edge of the box), and minimum and maximum values (horizontal lines). Points at a distance from the quartiles >1.5 times the inter-quartile range are plotted individually. Statistically significant p values between groups are reported (Kruskal-Wallis Rank Sum test).

As summarized in Figure 1A, in our previous study [16] the analysis of XCI and *XIST* RNA in human mammary epithelial cells (HMEC) and in human breast cancer cell lines revealed three distinct patterns: presence of both Xa and Xi and *XIST* expression (normal, type 0); Xa replication, Xi loss and no *XIST* expression (type 1); Xa replication, Xi loss and *XIST* expression (type 2). The latter finding is of particular interest since *XIST* is the only gene known to be exclusively expressed from the Xi under physiological conditions [3]. Interestingly, we found that all four cell lines not expressing *XIST* were *BRCA1* wild-type, whereas of the two cell lines positive for *XIST* expression in the absence of a Xi, one (HCC 1937) was *BRCA1* ^−/−^ (Figure 1A). Subsequent studies identified *XIST* expression in two additional *BRCA1* ^−/−^ cell lines (MDA MB 436 and L56Br-C1) [25], [26]. These findings, although indicating that *XIST* expression is not necessarily related to *BRCA1* status, prompted us to speculate on a possible role of *BRCA1* on *XIST* regulation.

To support this hypothesis we investigated, by quantitative RT-PCR, *XIST* RNA levels in two groups of BLC (9 *BRCA1*-related and 10 sporadic) compared to sporadic non-BLC (n = 11) (Figure 1B). In accordance with the observations by Richardson et al. (2006), who reported a higher frequency of Xi loss and Xa replication in BLC respect to non-BLCs, sporadic BLC exhibited a significant lower *XIST* expression than sporadic non-BLC (p = 0.0002). However, such reduced expression was not apparent in *BRCA1*-related BLC, that showed *XIST* RNA levels comparable to those of non-BLC (p = 0.23) and significantly higher than those detected in sporadic BLC (p = 0.004).

### BRCA1 knockdown leads to an enhanced *XIST* expression in cells with atypical XCI status

**Figure 2:**
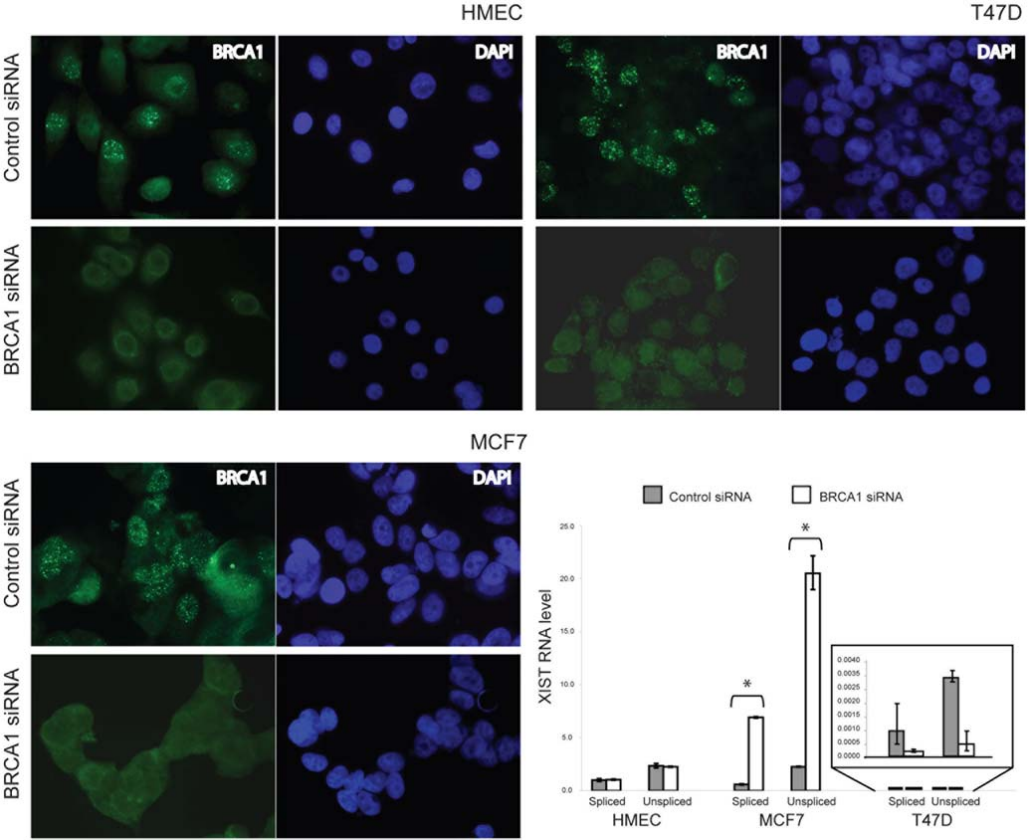
Effects of *BRCA1* RNAi on *XIST* expression in cells with different XCI status. HMEC (XCI type 0), MCF7 (XCI type 1) and T47D (XCI type 2) were transfected with a mix of two *BRCA1*-specific siRNAs, mapping to exons 12 and 24, or a control siRNA. After 72 hrs, cells were processed for BRCA1 immunofluorescence and RNA purification. In all panels BRCA1 is immunostained in green and nuclei are marked with DAPI. The histogram represents quantitative RT-PCR analysis performed on cDNAs of the indicated cell lines, before and after *BRCA1* silencing, using primers specific for spliced and unspliced *XIST* RNA. *XIST* RNA levels are expressed as a ratio to *GAPDH* mRNA levels, after subtraction of the background signal from cDNA synthesis reactions lacking reverse transcriptase. To facilitate comparison between cell lines with different XCI status, the *XIST*/*GAPDH* transcript ratio was normalised relative to HMEC. Error bars represent standard deviation and the asterisks indicate statistically significant differences (p<0.05, Student's test).

To verify the hypothesis that the lack of functional BRCA1 is involved in the inappropriate expression of *XIST* from Xa, we performed *BRCA1* silencing in HMEC (XCI-type 0) and in the following *BRCA1* ^wt^ breast cancer cell lines: MDA MB 231(XCI-type 1), T47D (XCI-type 1) and MCF7 (XCI-type 2). For RNAi-mediated BRCA1 knockdown we used a mix of two dsRNAs, mapping to exons 12 and 24. By immunofluorescence, we observed a complete protein depletion in all cell lines (Figures 2 and S1A). The impact of such acute BRCA1 knockdown on *XIST* expression was examined by real-time RT-PCR. The transient BRCA1-deficit did not affect *XIST* expression in type 0 and type 1 cells, whereas in type 2 cells we observed a significant increase of XIST levels, both for the spliced and unspliced RNA forms (∼12 times, P<0.05) (Figures 2 and S1A).

We verified whether the expression changes in MCF7 could be related to epigenetic modifications of the *XIST* promoter region. After BRCA1 knockdown, the pyrosequencing quantitative analysis of *XIST* CpGs showed demethylation (88% average methylation in control siRNA *vs* 67% in BRCA1 siRNA) (Figure S1B). In HMEC the analysis showed approximately 44% of methylation both before and after BRCA1 siRNA (Figure S1B).

MCF7 cell line is characterized by the presence of both XIST positive and negative sub-populations [25], [26]. Thus, the increase of XIST levels after BRCA1 silencing in this cell line might be due to a selection of XIST-positive cells, rather than the release of *XIST* regulation on Xa. However, subsequent experiments ruled out this possibility (see paragraph “*XIST* RNA staining persists with the same features after *BRCA1* silencing”). The overall results indicate a regulatory role of BRCA1 on Xa *XIST* allele.

The availability of experimental models differing for XCI type, in which we transiently repressed the expression of *BRCA1*, prompted us to further investigate the role of BRCA1 on XIST localization.

### Misbehaviour of *XIST* RNA in MCF7 cells

We evaluated the nuclear distribution of *XIST* RNA in MCF7 cells compared to HMEC.

**Figure 3:**
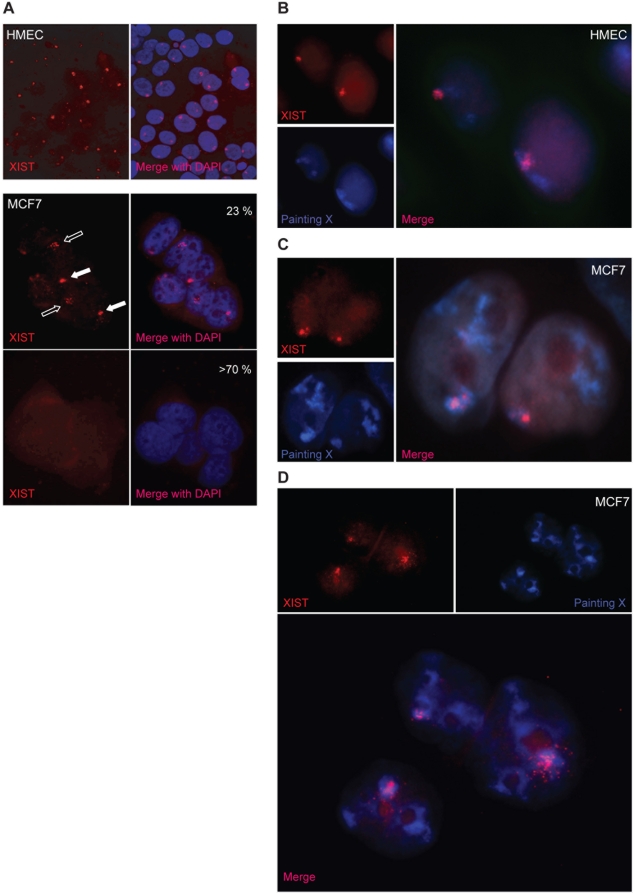
*XIST* RNA behaviour in HMEC and in MCF7 breast cancer cell line. A) Localisation of *XIST* RNA (red signal) revealed by RNA-FISH on HMEC and MCF7 nuclei (DAPI staining). The percentages of XIST-positive (middle) and XIST-negative (bottom) MCF7 nuclei are reported. Positive cells show different XIST distribution, clustered clouds (full arrows) or dispersed signals (empty arrows). B–D) Localisation of *XIST* RNA (red) respect to nuclear X chromosome territories (blue) revealed by FISH analysis in HMEC and MCF7 nuclei. MCF7 XIST-positive nuclei always show three X chromosome domains. In MCF7 nuclei the overlap between XIST and X chromosome territory appears more limited (panel C), respect to HMEC (panel B) and very often spreads outside the X chromosome domain (panel D).

First, we analyzed XIST X chromosome coating by RNA-FISH. Differently from normal XIST distribution observed in HMEC, *XIST* RNA staining in MCF7 showed an abnormal morphology, with a dispersed and mislocalized signal (Figure 3A), consistently with previous reports [25], [26]. Only about 32% of MCF7 nuclei were XIST-positive and among them 49.3% showed clustered (Figure 3A - full arrow) and 50.7% dispersed (Figure 3A – empty arrow) *XIST* RNA signals.

Next, we considered the *XIST* RNA localisation respect to the nuclear X chromosome territories. Combining X chromosome painting and XIST RNA-FISH, in HMEC we observed only a partial coating of nuclear Xi chromosome domain by *XIST* RNA (Figure 3B). This finding is in line with previous observations by Chadwick and Willard [28] indicating that the human Xi is packaged into at least two nonoverlapping heterochromatin types: one defined by the presence of *XIST* RNA, histone variant macroH2A, and histone H3 trimethylated at lysine 27 (H3TrimK27) and the other defined by H3 trimethylated at lysine 9, heterochromatin protein 1 (HP1) and histone 4 trimethylated at lysine 20. Differently, in MCF7 nuclei the overlapping of *XIST* RNA staining and X chromosome painting was more limited and the XIST signal often spread outside the X chromosome territory (Figures 3B and 3C). Therefore, in this tumor cell line XIST shows a misbehaviour, because it is expressed by an active X chromosome and does not stably coat the *XIST* expressing X chromosome.

### X chromosomes status in MCF7 cells

Based on the unusual XIST behaviour observed in MCF7 cells, we further characterized the status of X chromosomes in this cell line.

**Figure 4:**
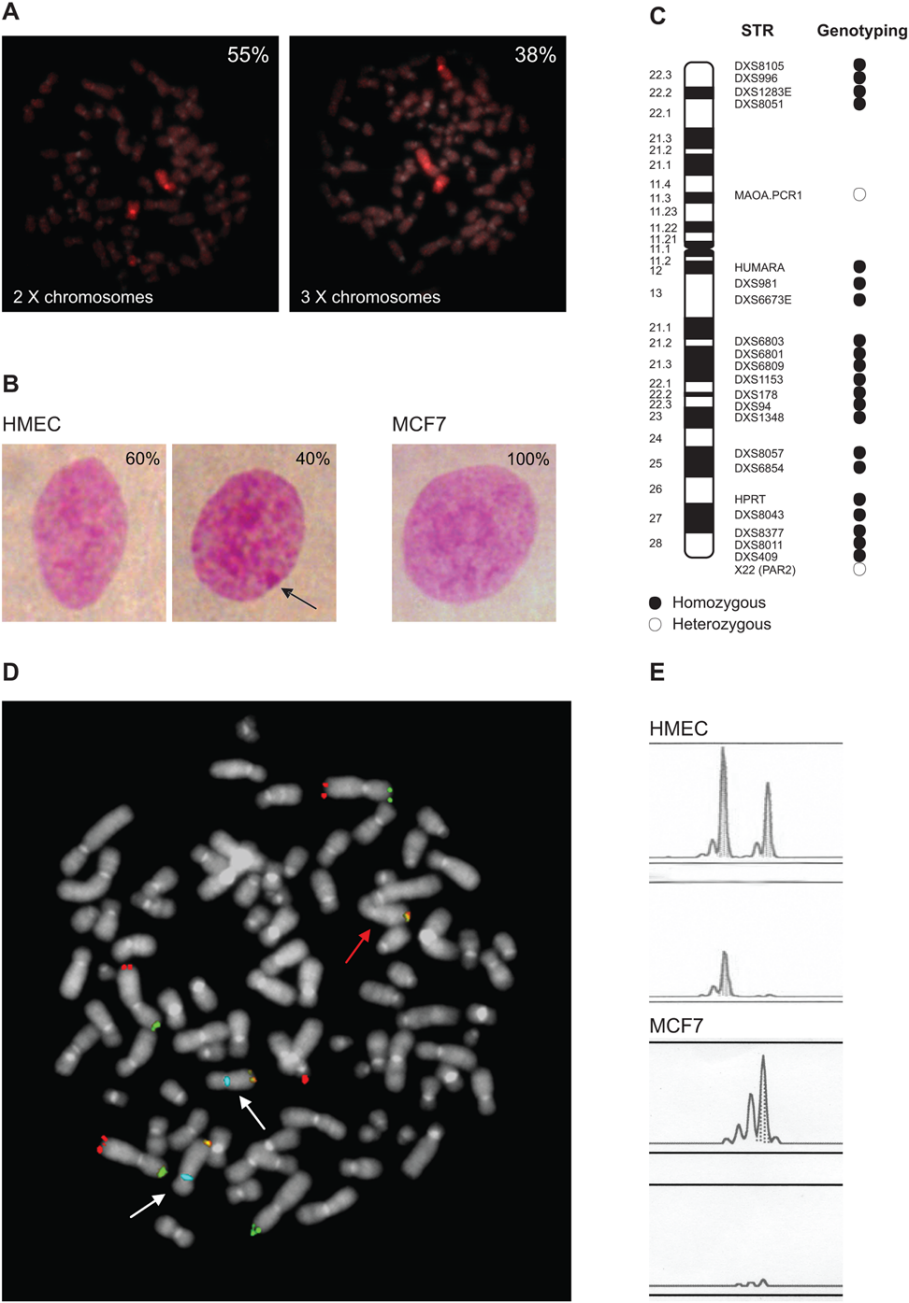
X chromosomes characterization of MCF7 cell line. A) FISH analysis on metaphases using X painting probe. The percentages of MCF7 cells with two (left) or three (right) X chromosomes are indicated. B) Barr body staining. The percentages of HMEC, used as positive control, with (right) or without (left) a detectable Barr body (arrow) are indicated. No positive cell was observed in MCF7. C) High level of homozygosity (87%) detected by genotyping of the indicated panel of STRs. D) DNA FISH using a mix of telomeric (red, green and orange) and X alpha-satellite (blue) probes. The orange spots decorate the Xq telomeric region: two on X chromosomes, positive for X-alpha satellite probe (white arrows) and one on a rearranged chromosome, negative for X-alpha-satellite probe (red arrow). Telomeric red and green probes were used as hybridization control. E) Methylation analysis of *ZMYM3* gene subjected to XCI (DXS6673E *locus*). Electropherograms show the allelic patterns obtained by PCR of HMEC and MCF7 DNAs undigested (top) and digested with methylation-sensitive enzymes (bottom). After digestion, HMEC show a methylated allele, whereas in MCF7 the *locus* is completely demethylated, resulting in the lack of amplification.

First, we assessed X chromosomes numbering by FISH, using both alpha-satellite and X painting probes. MCF7 cell line displayed two major subpopulations carrying two (∼55%) or three (∼38%) X chromosomes, respectively (Figure 4A).

Then, we performed a cytological evaluation of Xi by Barr body staining. In MCF7 cell line, we did not detect the Barr body in more than 700 examined nuclei; whereas, HMEC showed this cytological marker in approximately 40% of nuclei (Figure 4B).

X chromosome genotyping by Quantitative Fluorescence-PCR using 23 highly informative STRs demonstrated high levels of homozygosity (20/23, 87%), mainly in the q arm (17/18, 94%) (Figure 4C), whereas in HMEC homozygosity was about 38% [16 and data not shown]. Spots of heterozygosity in MCF7 were possibly due to chromosomal rearrangements, leading to the maintenance of residual segments of the homologous X chromosome, as shown by FISH using Xq telomeric probe (Figure 4D).

The observation of high level of homozygosity, together with the presence of two or more X chromosomes in the absence of detectable Barr body, is consistent with the duplication of the Xa and the loss of the inactive one. This is in keeping with the complete demethylation observed in MCF7 cells of X-linked genes subjected to XCI, including *AR*, *PGK1*, *POLA*, *OCRL* [16] and *ZMYM3* (Figure 4E).

These overall results ultimately demonstrate that in all the subpopulations present in MCF7 the native Xi is lost and all X chromosomes are copies of the native Xa, reinforcing our previous observations [16].

Finally, we evaluated *XIST* RNA origin by combined DNA and RNA FISH. We found that the MCF7 *XIST*-expressing population always displayed three X chromosomes, only one of which colocalized with the XIST signal (Figures 3B and 3C). Indeed, the frequency of *XIST*-expressing cells (32%) approached that of cells with three X chromosomes (38%), being the slight difference most likely due the difficulty in scoring XIST positive nuclei with a very dispersed signal.

### *XIST* RNA decorates a transcriptionally competent X in MCF7 cells

**Figure 5:**
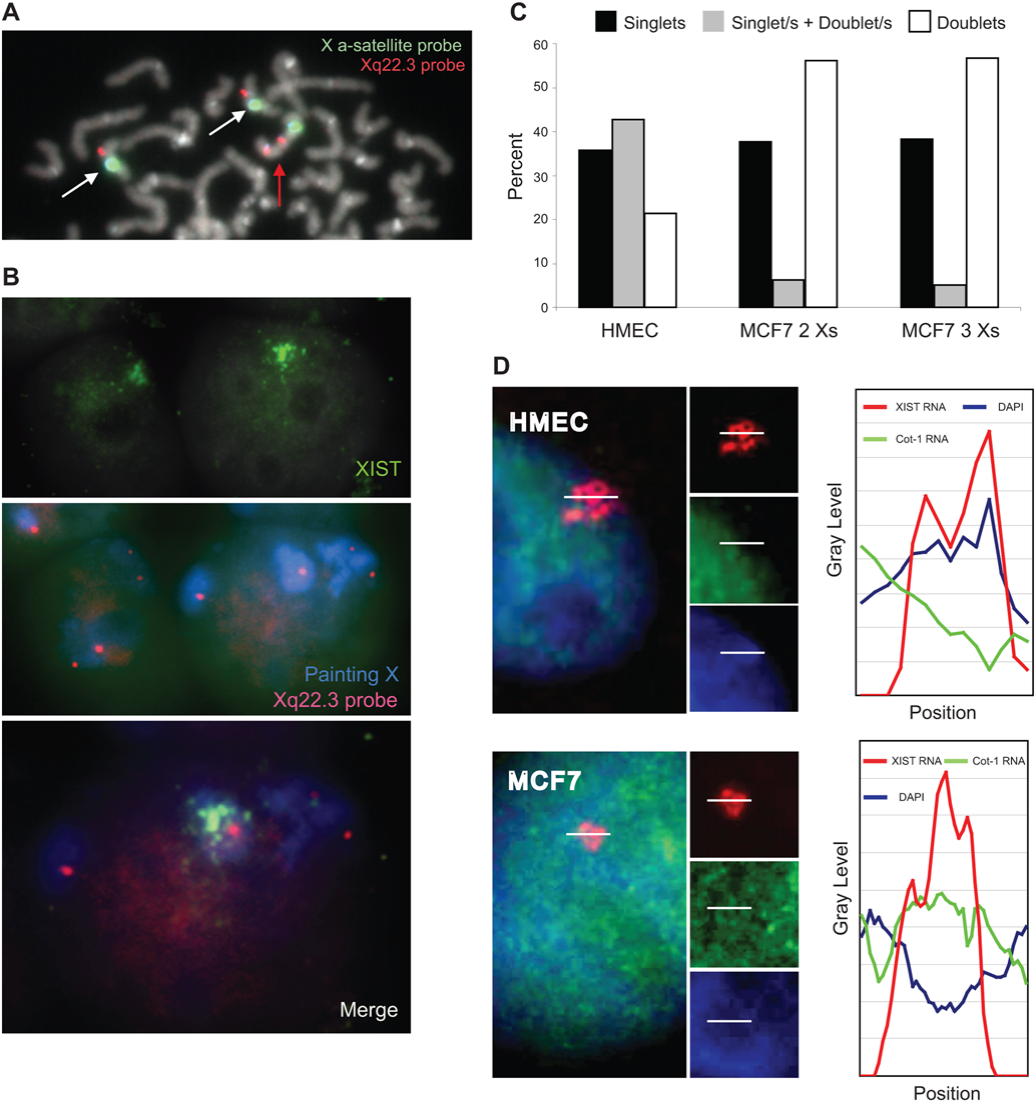
*XIST* RNA cloud paints an active X chromosome in MCF7 nuclei. A) DNA FISH using the single Xq22.3 locus RP11-349A16 probe (red), and X alpha-satellite probe (green). Cell populations with three Xs display two different hybridization patterns: two Xs with a single red spot (white arrows) and a chromosome with two red signals (red arrow). B) Simultaneous detection of *XIST* RNA (green), X chromosome territory (blue) and Xq22.3 locus (red). The X chromosome expressing XIST has one copy of the RP11-349A16 region (merge). C) Replication timing analysis. MCF7 and HMEC were briefly labelled with BrdU and analyzed by DNA FISH using the single Xq22.3 locus RP11-349A16 probe and by BrdU immunofluorescence. The pattern of FISH staining seen in BrdU-positive cells was scored for at least 300 nuclei of each cell type: nuclei with only “singlets” are those in which no Xs has yet replicated; nuclei with “singlet/s+doublet/s” pattern contain unreplicated and replicated Xs; nuclei with only “doublets” have all replicated Xs. Both MCF7 subpopulations with two and three Xs display a synchronous replication timing. D) Characterisation of the chromatin signatures of XIST-positive X chromosome in MCF7 and HMEC, by simultaneous FISH detection of *XIST* RNA (red) and Cot-1 RNA (green); DAPI nuclear staining is in blue. A line scan of fluorescence intensity (white bars) is shown for both cell types. In HMEC, the scan plot revealed overlap of the DAPI and *XIST* RNA signals, whereas the Cot-1 RNA signal is depleted, as expected for an inactive X chromosome. On the contrary, in MCF7 cells the line scan through the XIST-positive territory shows high intensity of the Cot-1 RNA signal combined with low DAPI intensity, typical signs of euchromatin.

To prove that in MCF7 the XIST-positive X chromosome resembled the features of the open chromatin, this was epigenetically and transcriptionally characterized. We targeted the *XIST* expressing chromosome by DNA/RNA FISH using the following probes: genomic single locus RP11-349A16 (Xq22.3), X painting and XIST. We took advantage on the localization of the RP11-349A16 probe within a cytogenetically evidenced interstitial duplication of Xq involving one of the X chromosome (data not shown). In the cell population with three X chromosomes, two Xs were visualized with a single RP11-349A16 signal and one with two signals (Figure 5A). As shown in Figure 5B, DNA/RNA FISH revealed that the XIST-positive X chromosome was one of two Xs showing a single RP11-349A16 signal.

Given that a shift of replication timing to late in S phase is a typical feature of Xi [1], [29], we performed FISH assay using RP11-349A16 probe on BrdU labelled cells, to verify asynchronous replication of X chromosomes. Both the cell populations characterized by two and three Xs showed synchronous X replication timing (Figure 5C), without any differences between Xs with single or double RP11-349A16 spots, whereas HMEC displayed a normal asynchronous X chromosome replication timing pattern (Figure 5C).

To completely characterize the chromatin signatures of the X chromosomes, we assessed the transcription/chromatin state of the XIST-positive X. The assay combined XIST staining and heterogeneous nuclear RNA (hsRNA) hybridization with Cot-1 DNA probe in non-denaturing conditions on DAPI stained nuclei. Cot-1 positive hsRNA is notably silent over the Xi and is detected throughout the Xa, whereas DAPI exhibits a higher affinity for heterochromatic regions [30]. In the vast majority of HMEC nuclei, a line scan throughout the XIST-positive X showed that the increase of the intensity of the *XIST* RNA signal had the same trend of DAPI, whereas Cot-1 RNA signal was depleted (Figure 5D). This picture is distinctive of the nucleoplasm territory of the normal heterochromatic inactive X. Conversely, in MCF7 cells the XIST-positive X chromosome displayed overlapping of increased Cot-1 RNA and *XIST* RNA signal intensities, associated with a reduced DAPI staining (Figure 5D), a typical sign of euchromatin and Xa (Clemson et al., 2006). Therefore, in MCF7 the colocalization of *XIST* RNA with an active chromatin domain, together with the absence of X chromosome asynchronous replication, revealed that XIST interacts with an euchromatic X. These unexpected findings bring to light that *XIST* RNA is able to decorate and to communicate also with an active X chromosome.

### *XIST* RNA staining persists with the same features after *BRCA1* silencing

To assess the function of BRCA1 in XIST localization, we monitored *XIST* RNA distribution before and after BRCA1 acute knockdown in cells with normal XCI state, in which *XIST* is expressed by Xi (HMEC), and in cells with abnormal XCI state, with *XIST* expressed by Xa (MCF7).

**Figure 6:**
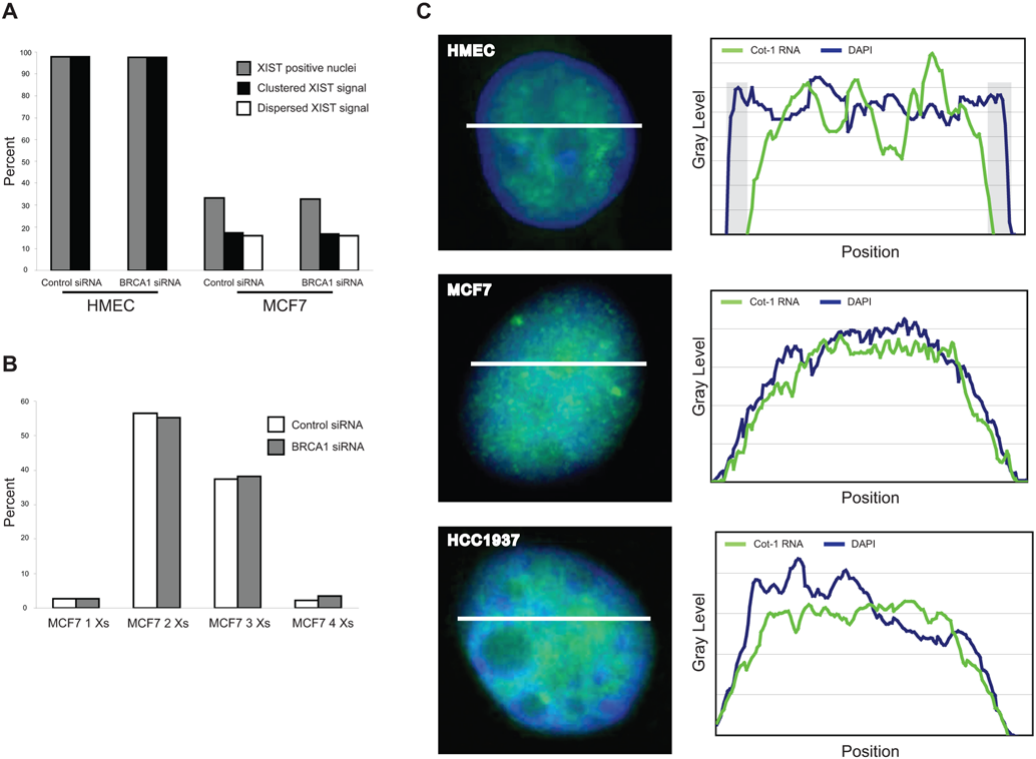
*XIST* RNA staining patterns and global defects of heterochromatin are independent of *BRCA1* status. A) Percentages of nuclei positive for *XIST* RNA signal before and after *BRCA1* silencing in HMEC and in MCF7. Specific signal morphology was evaluated, distinguishing clustered and dispersed signals. HMEC always display clustered staining only. In both cell types no relevant variation was observed after siRNA treatment. The results were reproducible in independent experiments. B) Distribution of MCF7 cell populations respect to X chromosome numbering before and after BRCA1 knockdown. No relevant variation in the relative content of the different populations was observed after siRNA treatment. The results were reproducible in independent experiments. C) Analysis of heterochromatin status in *BRCA1* normal and mutant cells by FISH analysis with Cot-1 probe (green) on DAPI-stained nuclei. A line scan of fluorescence intensity (white bars) is shown for each cell type. Shaded areas indicating regions of peripheral heterochromatin are evident in the scan plot relative to HMEC, but not in those of MCF7 (*BRCA1^wt^*) and HCC1937 (*BRCA1^−/−^*) breast cancer cell lines.

In HMEC, *XIST* RNA staining pattern in BRCA1 siRNAs-transfected cells was indistinguishable from that observed in non-specific siRNA-transfected cells, with an average of 97% of cells displaying a normal *XIST* RNA signal (Figure 6A). Likewise, in MCF7 cells the fraction of XIST-positive did not substantially change after *BRCA1* silencing (33% before vs 32% after silencing) (Figure 6A) and no difference in *XIST* RNA staining morphology was observed. In fact, the proportion of nuclei with dispersed and clustered signals was similar before and after BRCA1 knockdown (Figure 6A). Our data are consistent with previous reports [25], [26] and support the independence from BRCA1 of *XIST* RNA coating on the X chromosome, also in presence of an altered XCI status (*i.e*, MCF7).

Finally, the observation that *BRCA1* silencing in MCF7 did not modify the percentage of XIST-positive cells (Figure 6A) and of cells carrying three X chromosomes (Figure 6B) allowed us to exclude that the increased *XIST* RNA levels after BRCA1 knockdown (see Figure 2) was caused by the selection of the XIST-expressing subpopulation.

### Broader defects in heterochromatin are independent of *BRCA1* status

BRCA1 has been implicated in chromatin remodelling [31], [32]. Accordingly, HCC1937 *BRCA1^−/−^* breast cancer cell line shows a broader compromise of the heterochromatic compartment [12].

To assess whether broader defects on heterochromatin are specifically associated with BRCA1-deficit, we evaluated the features of whole heterochromatin in two breast cancer cell lines with different *BRCA1* constitution, MCF7 (*BRCA1* ^wt^) and HCC1937 (*BRCA1* ^−/−^). The analysis was based on the staining pattern of the Cot-1 RNA fraction (hsRNA), which decorates transcriptionally active areas of DNA, using Cot-1 probe in non-denaturing conditions [33]. As expected in normal cells [12], HMEC had a prominent rim of heterochromatin at the nuclear periphery, whereas both MCF7 and HCC1937 lacked this heterochromatic feature (Figure 6C). This evidence indicates that defects in heterochromatin compartment are a trait of breast cancer cells, irrespective of *BRCA1* status.

## Discussion

Among the several roles of BRCA1, a possible interaction with *XIST* RNA has been speculated. This relationship could help explaining the gender related effect of deleterious *BRCA1* germline mutations. However, the conflicting evidences on a communication between XIST and BRCA1 [13], [15], [25], [26] prompted us to further investigate on this issue and on XIST behaviour in breast cancer cells.

We found significant higher levels of *XIST* RNA in *BRCA1*-associated, respect to sporadic BLC. In light of a previous report that Xi loss and replication of the native Xa are common features in BLC [17], these observations suggest an influence of BRCA1 in regulating *XIS*T allele on Xa. Indeed, the positive effect of BRCA1 deficit on Xa *XIST* expression is demonstrated *in vitro* by *BRCA1* silencing in XCI-type 2 (Xi negative/XIST positive) MCF7 breast cancer cell line, which leads to a significant increase of XIST levels and promoter demethylation. This can be due to the release of BRCA1-mediated *XIST* regulation on Xa. The same phenomenon could occur also *in vivo*, where, however, the situation appears more complex, since BRCA1 tumors show heterogeneity of XCI status, caused by genetic instability that can lead not only to the loss but also to the gain of Xi copies [27]. Irrespective of the precise mechanisms involved, the different *XIST* RNA levels in *BRCA1*-related vs. sporadic BLC support the interest in investigating *XIST* expression as a possible marker to distinguish between these two groups of tumors. Noticeably, the modification of XIST expression induced by *BRCA1* silencing in XCI-type 2 cells is not detected in type 0 and type 1 cell lines, where Xa *XIST* promoter is completely repressed [16], thus suggesting that BRCA1 transient knockdown has no effect on a silenced Xa promoter.

Herein, we provide additional evidences that in MCF7 the Xi is lost and all X chromosomes are copies of the native Xa, as shown by the widespread homozygosity of X-linked STRs. The epigenetic and transcriptional characterization of MCF7 XIST-positive X chromosome shows active transcription and absence of heterochromatization. Moreover, synchronous replication timing of all X chromosomes is observed. All above findings are signatures of an active X.

The unusual discovery of *XIST* expression in a cell line carrying only native Xa copies prompted us to verify the nuclear distribution of *XIST* RNA respect to the X chromosome domains in MCF7 cells. We found a misbehaviour of *XIST* product manifested as a limited and unstable coating on the X chromosome. In light of this observation, we argue that *XIST* RNA cloud in the nucleus does not prove per itself the presence of an inactive X chromosome and conclude that the mislocalization of XIST cannot be considered as an indirect evidence of compromised Xi heterochromatin, in contrast to what suggested by Pageau et al. [12].

We found that depletion of BRCA1 in both HMEC and MCF7 does not appreciably modify XIST nuclear signal morphology. The maintenance of the XIST features after BRCA1 knockdown in HMEC is in keeping with previous observations from independent studies [25], [26] and indicate that BRCA1 is not the main actor driving XIST on X chromosome. This is corroborated by the analysis of *XIST* RNA signal morphology in MCF7 cells with an atypical *XIST* expression, which showed overlapping patterns before and after *BRCA1* silencing. Consequently, our findings suggest a reconsideration of those conclusions of previous studies on the communication between XIST and BRCA1 [12], [13], [15], [17] that were based on the incorrect assumption that the detection of the nuclear XIST cloud in tumor cells is necessarily indicative of the presence of the native Xi.

The analysis of the nuclear distribution of XIST in normal cells revealed a partial coating of interphase Xi chromosome domain. This finding is in keeping with the observations of Chadwick and Willard [28] that showed *XIST* RNA association exclusively with H3TrimK27-defined heterochromatin, and not with the other spatially distinct type of Xi heterochromatin. Given that H3TrimK27 is a peculiar feature of Xi [28], this might explain why in Xi negative MCF7 cells *XIST* RNA disperses in the nucleus. Intriguingly, one may speculate that, in the absence of its physiological targets, XIST could be attracted by H3TrimK27 domains outside the Xi, modifying the epigenetic status of such regions and possibly deregulating *in trans* the expression of tumorigenesis related loci.

Alterations of XCI status in cancer cells should be considered in the context of the overall chromatin organization. Studies of the cancer epigenome often reveal changes of chromatin status, as well as global hypomethylation and histone deacetylation [34]. Pageau et al. [12] speculated on a possible involvement of BRCA1 on chromatin pattern, given its association with constitutive heterochromatin-rich structures [31], [32]. The Authors reported genome-wide deficit in heterochromatin maintenance in HCC1937 *BRCA1^−/−^* breast cancer cell line. However, we found that global heterochromatin defects are present in breast cancer cell lines independently of *BRCA1* status. Our data are in keeping with previous findings [34], indicating the presence of epigenome defects as a common feature of tumor cells, rather than a unique association between BRCA1 and global heterochromatin maintenance.

In conclusion, our study provides further evidence that BRCA1 is not involved in XIST localization and demonstrate that the detection of XIST in a cancer cell is not indicative of the presence of an inactive X chromosome. However, the observation of inappropriate XIST expression/localization in cancer cells sheds light on a possible new mechanism of breast carcinogenesis. This mechanism might apply in particular to *BRCA1*-related cancer given the observed role of BRCA1 on the regulation of *XIST* expression from the Xa.

## Materials and Methods

### Ethics statement

All patients whose biological samples were included in the study signed an informed consent, approved by the Independent Ethical Committee of Istituto Nazionale dei Tumori, Milano (Italy), to donate to the Istituto Nazionale Tumori the leftover tissue specimens after completing diagnostic procedures for research purposes.

### Materials

Breast cancer cell lines MCF7, MDA MB 231, T47D and HCC1937 and normal human epithelial mammary cells (HMEC) were maintained as previously reported [16].

Frozen primary human breast carcinomas from patients with constitutional mutations of *BRCA1* and sporadic cases were retrieved from the Biobank of the Istituto Nazionale Tumori. The presence of *BRCA1* mutations was ascertained as previously reported [16], whereas sporadic cases were selected based on negative family history of cancer and age of onset >40 years. Classification of cancer samples as BLC was based on the simultaneous negativity for the expression of estrogen receptor (ER), progesterone receptor (PR) and HER2/Neu protein assessed as described [35].

### Interphase indirect immunofluorescence

Cells were grown in chamber slides (LAbTek II) for 48–72 hours, rinsed with PBS, fixed and permeabilized with 4% paraformaldehyde, 0,1% Triton X-100 for 15 min at RT and blocked with PBS, 0,1% Triton X-100 and 5% BSA for 30 min at RT. Cells were then incubated with primary anti-BRCA1 policlonal antibody (dilution 1∶50, ID# 9010 Cell Signalling) overnight at 4°C in a humidified chamber, and subsequently washed three times for 5 min in PBS. A FITC anti-rabbit secondary antibody (dilution 1∶200, Sigma) was then applied for 2 hours at RT in a humidified chamber. Slides were washed three times in PBS and counterstained with DAPI-antifade (Vector laboratories).

### RNA FISH

Cells were grown in chamber slides, briefly washed with both Hank's balanced salt solution (Euroclone) and CSK Buffer (10 mM NaCl, 300 mM Sucrose, 3 mM MgCl_2_, 10 mM PIPES pH = 6,8); all steps were done on ice and solutions were prepared with DEPC water. Slides were fixed with 4% paraformaldehyde for 8–10 min at 4°C, treated with a solution containing CSK Buffer, 0,5% Triton×100, 2 mM VRC (vanadyl ribonucleoside complex, Sigma) for 10–12 min at 4°C, and incubated in 70% ethanol for 10 min at 4°C. They were subsequently dehydrated in 70%, 85% and 100% ethanol for 5 min each at −20°C and finally air dried. XIST probe was obtained by Long Range PCR (Long Range PCR-Kit Expand 20 Kb PLUS PCR System – Roche) amplifying exons 1 and 6 of *XIST* gene and pulling them together. PCR primers were as following: exon 1 sense: 5′ CCCAGCTTCTCTCGAAAGTCACTCTAAT 3′; exon 1 antisense: 5′ AGTGAAGGC TTATCCACCTAGTTCAGGC 3′; exon 6 sense: 5′ ATTCTCTCTCCTCCCCTGCGT 3′; exon 6 antisense: 5′TGGTAGTGATGCCAGAAACTGTGA 3′. PCR products were labeled by random priming with Cy3-dUTP (Prime-It Fluor, Fluorescence labeling Kit – Stratagene) following the provided protocol. Cot-1 probe was obtained labeling 100 ng of Cot-1 DNA (1 mg/ml, Invitrogen) by Prime-It Fluor, Fluorescence labeling Kit with FITC-dUTP. The probes were then ethanol precipitated, washed in 70% ethanol, air dried and resuspended in 15 µL of hybridization buffer (50% formamide, 4× SSC, 20% dextran sulfate, 40 mM VRC, 0,4% BSA). The probes were finally denatured at 76°C for 10 min before overnight hybridization at 37°C. The slides were then washed with 50% formamide/2× SSC for 3 times at 42°C for 5 min each, and with 2× SSC for 3 times at 42°C for 5 min each and mounted with DAPI-antifade (Vectashield).

Image analysis was performed using Soft Imaging System Cell, Olympus Cell Family.

### DNA-FISH

DNA-FISH on cells grown in chamber slides after RNA-FISH protocol was performed as follow. Slides were washed in PBS, treated with 4% paraformaldehyde, 0,1% Triton×100 in PBS for 10 min at RT. They were subsequently dehydrated in 70%, 85%, 100% ethanol for 5 min each at RT, air dried and hybridized using the following probes: BAC RP11-349A16 (Xq22.3) (UCSC Genome Database, http://genome.ucsc.edu/ ), X chromosome alpha-satellite (Kreatech) and X chromosome painting (WCP, Cambio). The latter probe was Biotin labelled and detected by DEAC-streptavidin; BAC RP11-349A16 probe was labeled by Nick-Translation Kit (Roche). DNA FISH conditions were as reported by CAMBIO instructions.

DNA FISH was also performed on MCF7 chromosome preparations using BAC probe RP11-349A16 and X chromosome alpha-satellite, according to Lichter et al. [36] with minor modifications and using Mix1 and Mix 2 ToTelVysion Multi-color DNA Probe (Vysis) and X chromosome alpha-satellite, according to Vysis instructions.

### Replication timing assay

Replication timing analysis for X chromosome was performed using the BAC probe RP11-349A16 as previously described [37].

### RNA interference

BRCA1 knockdown was obtained by RNA interference. A mix of two different dsRNAs was used: BRCA1-A (ID#5479, Ambion) mapped to exon 12 and BRCA1-B, designed with Cenix Designed siRNAs program (Ambion, www.ambion.com/siRNA), mapped to exon 24. The BRCA1-B siRNA sequence was: 5′ GGUUUCUUAAAGUCUGAGA 3′.

*BRCA1* dsRNAs were co-transfected (100 pmol BRCA1-A and 60 pmol BRCA1-B) into cell lines by reverse transfection method, using Lipofectamine RNAiMAX (Invitrogen). Briefly, a transfection mix, containing Lipofectamine RNAiMAX was resupended in the growth medium, incubated for 20 min RT. Cells to be transfected were harvested with 0.25% trypsin solution and 2.5×10^4^ cells were added to the transfection mix and maintained in culture for 72 hours. The Non-specific Control Pool (Dharmacon), dsRNAs without homology to human-specific transcripts, was used as a negative control.

RNAi experiments were performed on an unique pull of cells, splitted in different plates for immunofluorescence, RNA and DNA-FISH, methylation assay and Real-Time RT-PCR.

### Barr Body staining

Cells were grown in chamber slides for 3 or 4 days, rinsed with 0.9% NaCl and fixed with ethanol/acetic acid (3∶1v/v). Barr body staining was performed as previously reported [38].

### Real-Time RT-PCR

RNA was isolated from cultured cells using TRI-REAGENT (Total RNA Isolation Reagent, Sigma) following the manufacturer's instructions, eliminating a possible genomic DNA contamination by DNA Free Kit (Ambion). 500 ng of total RNA were retro-transcribed using the Super Script™ III Platinum (Two-Step qRT-PCR Kit, Invitrogen) and the obtained cDNA was used as template for quantitative Real-Time PCR, based on TaqMan methodology, using the ABI PRISM 7500 Fast Sequence Detection System (Applied Biosystems). The amount of *XIST* RNA was calculated using the 2^−ΔΔCt^ method relative to *GAPDH* housekeeping gene, selected from a pool of tested housekeeping genes, because it showed the same amplification efficiency in a scale of RNA concentration. Primers and probes for both *XIST* and *GAPDH* were provided by Applied Biosystems (TaqMan® Gene Expression Assay, ID#: Hs00300535_s1 unspliced XIST, Hs01079824_m1 spliced XIST and 4333764 spliced GAPDH). The experiment was also performed on untranslated RNA to verify possibile DNA contamination.

For primary human breast carcinomas, total RNA was extracted using Trizol reagent (Life Technologies) following the manufacturer's instructions and treated with DNaseI (Qiagen). RNA was reverse-transcribed using the High-Capacity cDNA Archive Kit (Applied Biosystems) and the expression levels of *XIST* were analyzed on an ABI PRISM 7700 instrument (Applied Biosystems) using a specific TaqMan® Gene Expression Assay (ID# Hs01079824_m1) and the TaqMan® Pre-Developed Assay for the 18S ribosomal RNA housekeeping gene (part no. 4319413E) for normalization. Data were analyzed using the Sequence Detector v1.9 software, and statistical analyses were performed using the R-statistical computing programming language (R Development Core Team 2006 R: a language and environment for statistical computing. R Foundation for Statistical Computing, Vienna, Austria. ISBN 3 900051- 07-0, URL http://www.R-project.org.). Gene expression data were quantified as described by the manufacturer and log2-transformed to obtain normally distributed values. The log2-transformed expression was interpreted as the difference between the observed threshold cycle (Ct) of the reference gene and that of the gene of interest. Since in our analysis the housekeeping gene was found to be expressed at a higher level than XIST, the computed ΔCt values resulted negative.

### X chromosome genotyping

DNA was extracted from cultured cells using standard phenol-chloroform-isoamylic method. X chromosome was genotyped for the following STRs (Short Tandem Repeats) markers, selected from the National Center for Biotechnology Information (http://www.ncbi.nlm.nih.gov/): DXS8105, DXS996, DXS1283E, DXS8051, MAOA.PCR1, HUMARA, DXS981, DXS6673E, DXS6803, DXS6801, DXS6809, DXS1153, DXS178, DXS94, DXS1348, DXS8057, DXS6854, HPRT, DXS8043, DXS8377, DXS8011, DXS409 and Xp22. They were individually amplified using FAM-labelled primers and the PCR products were run on Fluorescent Capillary Systems ABI PRISM 310/3130 (Applied Biosystem) and analyzed with GeneScan and GeneMapper Softwares. The alleles analysis was carried out following the criteria of QF-PCR [39] in order to detect the presence of possible low levels of heterozygosity, related to cell population heterogeneity. The standard range of the peak's area values ratio was previously reported in a subset of STRs of our panel [39] or, for the other markers, it was home-made set up evaluating allele ratio from 20 voluntary donors healthy females with an informative/heterozygous allelic pattern, calculating the short to the long area peak ratio.

### Methylation assay

To quantify the methylation levels of *XIST* promoter we analyzed a region previously described as methylated on Xi chromosome [40], using Pyrosequencing technology.

The bisulphite conversion of genomic DNA (1 µg) was obtained using EZ DNA methylation kit (Zymo Research). After bisulphite treatment, PCR was carried out in a final volume of 50 µl with 2.5 unit of Promega Go-Taq polymerase (Promega). The primers for modified sequences were: sense: 5′-TTGATTATTTGGTGGTGTGTGAG-3′ (gi:37704377, 832-854) and biotinylated antisense: 5′-TCATCCATCTTACCTCCCTAATTT–3′ (gi:37704377, 1009-986). The PCR conditions were 40 cycles of 95°C for 30 sec, 50°C for 30 sec and 72°C for 20 sec, followed by 72°C for 5 min. 40 µl of PCR product were used for pyrosequencing assay using the sequencing primer: 5′-TTTAGATTGTGGAGGAAAAG-3′ (gi:37704377, 906-925).

Pyrosequencing reactions were performed in the PSQ HS 96 System (Biotage), using Pyro Gold Reagent kits (Biotage). Methylation was quantified using Pyro Q-CpG Software (Biotage) that calculates the ratio of converted C's (T's) to unconverted C's at each CpG, giving a percentage of methylation.

The methylation status of promoter region of *ZMYM3* X-linked gene subjected to XCI (DXS6673E *locus*) was analyzed by PCR using as template the genomic DNA previously digested with the methylation sensitive enzymes HhaI and RsaI (New England Biolabs). Primers and method are fully described in Beever et al. [41].

## Supporting Information

Figure S1 Effects of *BRCA1* RNAi on *XIST* expression and promoter methylation status in breast cancer cell lines. A) Effects of *BRCA1* RNAi on *XIST* expression in MDA MB 231(XCI type 2) breast cancer cell line. Cells transfected with a mix of two *BRCA1*-specific siRNAs, mapping to exons 12 and 24, or a control siRNA. After a 72 hrs, cells were processed for BRCA1 immunofluorescence or RNA purification. BRCA1 is immunostained in green and nuclei are marked with DAPI. The histogram represents quantitative RT-PCR analysis performed on cDNA before and after *BRCA1* silencing, using primers specific for spliced and unspliced *XIST* RNA. XIST levels are expressed as a ratio to *GAPDH* mRNA levels after subtraction of background signal from cDNA synthesis reactions lacking reverse transcriptase. To facilitate comparison between cell lines with different XCI status, the ratio of *XIST∶GAPDH* transcripts was normalised relative to normal HMEC. Error bars represent standard deviation. B) Assessment of *XIST* promoter methylation levels, by pyrosequencing, in HMEC and MCF7. The percentages of methylation at four CpG positions before and after *BRCA1* silencing are reported. In brackets the mean methylation is indicated. The MCF7 promoter shows demethylation after BRCA1 silencing.
(5.88 MB EPS)
